# MiR-9, miR-21, and miR-155 as potential biomarkers for HPV positive and negative cervical cancer

**DOI:** 10.1186/s12885-017-3642-5

**Published:** 2017-09-21

**Authors:** Sunyoung Park, Kiyoon Eom, Jungho Kim, Hyeeun Bang, Hye-young Wang, Sungwoo Ahn, Geehyuk Kim, Hyoungsoon Jang, Sunghyun Kim, Dongsup Lee, Kwang Hwa Park, Hyeyoung Lee

**Affiliations:** 1 Department of Biomedical Laboratory Science, College of Health Sciences, Yonsei University, Wonju-si, Gangwon-do 26493 Republic of Korea; 2 Optipharm M&D, Inc., Wonju Eco Environmental Technology Center, Wonju-si, Gangwon-do 26493 Republic of Korea; 3 Department of Clinical Laboratory Science, College of Health Sciences, Catholic University of Pusan, Pusan, Republic of Korea; 4 Department of Clinical Laboratory Science, Hyejeon College, Hongseoung, Republic of Korea; 5 Department of Pathology, Wonju College of Medicine, Yonsei University Wonju College of Medicine, 20 Ilsan-ro, Wonju-si, Gangwon-do 26426 Republic of Korea

**Keywords:** Cervical cancer, microRNA, HPV E6/E7, RT-qPCR, Molecular diagnosis

## Abstract

**Background:**

Cervical cancer is the second leading cause of death among female patients with cancer in the world. High risk human papillomavirus has causal roles in cervical cancer initiation and progression by deregulating several cellular processes. However, HPV infection is not sufficient for cervical carcinoma development. Therefore, other genetic and epigenetic factors may be involved in this complex disease, and the identification of which may lead to better diagnosis and treatment. Our aim was to analyze the expression of microRNAs in cervical cancer cases positive or negative for HPV E6/E7 mRNA, and to assess their diagnostic usefulness and relevance.

**Methods:**

The expression of three different microRNAs (miR-9, miR-21, and miR-155) in 52 formalin-fixed paraffin-embedded (FFPE) primary cervical cancer tissue samples and 50 FFPE normal cervical tissue samples were evaluated.

**Results:**

MiR-9, miR-21, and miR-155 were significantly overexpressed in cervical cancer tissues compared to normal tissues (*P* < 0.001). MiR-21 and miR-155 expression combined with the HPV E6/E7 mRNA assay in HPV E6/E7 negative cervical cancer showed increased AUC of 0.7267 and 0.7000, respectively (*P* = 0.01, *P* = 0.04), demonstrating their potential as diagnostic tools. Moreover, miR-21 and miR-155 were predictors showing a 7 fold and 10.3 fold higher risk for HPV E6/E7 negative patients with cervical cancer (*P* = 0.024 and *P* = 0.017, respectively) while miR-155 was a predictor showing a 27.9 fold higher risk for HPV E6/E7 positive patients with cervical cancer (*P <* 0.0001).

**Conclusions:**

There is a strong demand for additional, alternative molecular biomarkers for diagnosis and management of precancer patients. MiR-21 and miR-155 may be helpful in the prediction of both HPV positive and HPV negative cases of cervical cancer.

## Background

Cervical cancer is the third most common malignancy in women worldwide [1]. High risk human papillomavirus (HR-HPV) infection is recognized as the most important risk factor in cervical cancer. Persistent over-expression of the E6 and E7 oncogenes encoded in the HPV genome have a critical role in the development of cervical cancer by causing genetic and epigenetic instability [2]. HPV E6 leads to the degradation of p53, which is a critical tumor suppressor that regulates abrogation of cell growth arrest. Furthermore, HPV E7 binds and deactivates another important tumor suppressor, the retinoblastoma protein (pRb), thereby interfering with cell cycle regulation [3–6].

Recently, several studies reported the development of cervical cancers that are HPV negative despite increased sensitivity of HR-HPV detection methods. Through meta-analyses of HPV detection methods, both Tjalma, et al. and Giorgi, et al. found that 4.2 to 8.2% of cases were HPV negative in 574 invasive cervical cancers and 3162 invasive cervical cancers [7, 8]. A large international retrospective cross-sectional study including 10,575 cases with invasive cervical cancer found that 15% (1598 cases) were negative for HPV DNA [9]. Similarly, our previous study also found that 15% of patients with cervical cancer (100 cases) were HPV negative [10].

Epigenetic instability is affected by microRNAs (miRNA or miR-). MiRNAs are 19 to 25 nucleotides (nt) in length, and have a role in transcriptional and epigenetic regulation through binding the 3′-UTR of the target-mRNA [11, 12]. It is now widely known that miRNA dysregulation is associated with a wide variety of human malignancies, such as breast cancer, lung cancer, colon cancer, and gastric cancer [13–16].

Many miRNAs studies have tried to confirm the utility of each miRNAs in cervical cancers with different methods. Lui et al. used miRNA direct sequencing analysis with six human cervical carcinoma cell lines and frozen cervical tumor tissues [17]. Lee et al. had miRNA expression profiling with 157 panel analyses with frozen cervical tumor tissues [18]. Gocze et al. utilized quantitative real time polymerase chain reaction (RT-qPCR) of eight miRNAs (miR-21, miR-27a, miR-34a, miR-146a, miR-155, miR-196a, miR-203, miR-221) individually [19].

Among these several miRNAs, three miRNAs (miR-9, miR-21, and miR-155) having their revealed targets that might be related to cancer were selected. Ma et al. showed miR-9 increased cell motility and invasiveness by targeting Cadherin 1(CDH1) and lead to cancer metastasis [20]. Asangani et al. and Bumrungthai et al. showed miR-21 promoted invasion and cell proliferation targeting programmed cell death 4(PDCD4) [21, 22]. MiR-155 expression promotes the proliferation targeting liver kinase B1 (LKB1) [23, 24].

Although the roles of these three miRNAs (miR-9, miR-21, and miR-155) have been studied in cervical cancer, their potential diagnostic or prognostic value in a clinical setting has not been examined. In addition, it is not known whether there is an association between these three miRNAs and HR-HPV infection status in clinical tissue specimens. Therefore, the purpose of this study was to investigate miR-9, miR-21, and miR-155 expression levels in cervical cancer and normal tissue samples, and determine their possible relation to HR-HPV E6/E7 oncogene expression.

## Methods

### Clinical samples

A total of 52 FFPE cervical cancer tissue samples and 50 FFPE normal cervical tissue samples were used from the Department of Pathology, Yonsei University Wonju Severance Christian Hospital, Wonju, Republic of Korea, between January 2010 and December 2014 (Table 1). Institutional Ethics Committee at Yonsei University Wonju College of Medicine approved the study protocol (approval no. YWMR-12-4-010) and all subjects provided written informed consent. Cases with tissue biopsies available were reviewed by two pathologists. The 52 cervical cancer samples consisted of tissue samples from 50 squamous cell carcinomas and 2 adenocarcinomas.

**Table 1:** Sample information in cervical cancer and normal

Variables	Cancer, n (%)	Normal, n (%)
Age		
< 50 years	18 (34.6)	31 (62.0)
≥ 50 years	34 (65.4)	19 (38.0)
Histology		
SCC	50 (96.2)	
ADC	2 (3.8)	
HPV E6/E7 mRNA expression		
Positive	37 (71.2)	0 (0)
Negative	15 (28.8)	50 (100)
Total	52 (100)	50 (100)

*SCC* Squamous cell carcinoma, *ADC* Adenocarcinoma

### Deparaffinization of FFPE tissues and total RNA extraction

Three to four 10-μm thick sections of FFPE cervical tissue were used for total RNA extraction. To remove paraffin from FFPE tissue, 160 μL of Deparaffinization solution (Qiagen, Hilden, Germany) was added and vortexed, followed by incubation for 3 min at 56 °C. RNA extraction was performed using the Qiagen RNeasy FFPE kit (Qiagen, Hilden, Germany) according to the manufacturer’s protocol. Total RNA purity and concentration were determined by measuring the ratio of the absorbance at 260 and 280 nm using an Infinite 200 spectrophotometer (Tecan, Salzburg, Austria). All preparation and handling procedures were conducted under RNase-free conditions. Isolated total RNA was stored at −70 °C until used.

### cDNA synthesis

Complementary DNA (cDNA) was synthesized using a TaqMan microRNA Reverse Transcriptase kit (Applied Biosystems by Life Technologies, Foster City, CA, USA) according to manufacturer’s instructions. Briefly, 5 to 10 ng of total RNA was used for cDNA synthesis. The reverse transcriptase (RT) reaction mixture contained 0.15 μL of 100 mM dNTP mix (100 mM each dATP, dGTP, dCTP, and dTTP at a neutral pH), 1 μL of 50 U/μL reverse transcriptase, 1.5 μL of 10× reverse transcriptase buffer, 0.19 μL of 20 U/μL RNase inhibitor, and adjusted the total reaction volume to 15 μL with nuclease free water. The cDNA synthesis reaction was performed as follows: 16 °C for 30 min followed by 42 °C for 30 min, and 85 °C for 5 min.

### MiRNA analysis using RT-qPCR

MiRNA expression was quantified by determining the cycle threshold (C_T_) which is the number of PCR cycles required for the fluorescence to exceed a value significantly higher than the background fluorescence, using the TaqMan small RNA assay (Applied Biosystems by Life Technologies) with miRNA specific primers according to manufacturer’s instructions. Briefly, 1.4 μL of cDNA was added to 10 μL of probe qPCR mix and 7.6 μL of nuclease free water. The following TaqMan small RNA assay (Applied Biosystems) primers were used: hsa-miR-9-5p, hsa-miR-21-5p, hsa-miR-155-5p, and RNU6B. All analyzed miRNAs are of human (*Homo sapiens*) origin and therefore, the prefix “hsa” is omitted throughout the text. RT-qPCR reactions were performed using a CFX96 Real-Time PCR System Detector (Bio-Rad, Hercules, CA, USA). Samples were run in duplicate for each experiment. Data were analyzed using the comparative Ct (2-ΔΔC_T_) method using the small nuclear RNA, RNU6B, as an endogenous control. To monitor reagent contamination, negative controls were included for each primer pair. PCR cycling conditions were as follows: 95 °C for 3 min 40 cycles of 95 °C for 15 s and 60 °C for 60 s.

### HPV E6/E7 mRNA analysis using RT-qPCR

To detect HPV E6/E7 mRNA in FFPE cervical tissues, multiplex RT-qPCR was performed using the TaqMan assay with the OPTIMYGENE HPV E6/E7 mRNA RT-qDx assay kit (Optipharm, Osong, Republic of Korea). PCR primers and the corresponding TaqMan probes were designed for three different sets of HPV regions, with each set of probes targeting their conserved sequence (FAM: HPV genotypes 16, 31, 33, 35, 52, and 58; CY5: HPV genotypes 18, 39, 45, 51, 59, and 68; and HEX: HPV genotypes 53, 56, 66, and 69).

RT-qPCR reactions consisted of 10 μL of 2 × Thunderbird probe qPCR mix (Toyobo, Osaka, Japan), 5 μL of primers and TaqMan probe mixture, 2 μL of template cDNA, and distilled water for a final reaction volume of 20 μL. The multiplex RT-qPCR assay detected the HPV E6 and E7 genes simultaneously in a single tube by incorporating two targets (E6 and E7) using specific TaqMan probes, which were labeled with different fluorophores (FAM, HEX, and Cy5). Positive and negative controls were included throughout the procedure. PCR cycling conditions were as follows: 95 °C for 3 min 45 cycles of 95 °C for 20 s and 60 °C for 40 s. To avoid false negatives because of mRNA degradation, glyceraldehyde-3-phosphate dehydrogenase (GAPDH) was used as an endogenous control.

### Statistical analysis

Statistical analysis was performed using GraphPad Prism software version 5.02 (GraphPad, La Jolla, CA, USA) and MedCalc 9.0 software (MedCalc Software Inc., Mariakerke, Belgium). Student’s t-test and Mann Whitney *U* test were used to determine statistical significance between cervical cancer and normal cervical tissue samples as well as investigate miRNA expression in patients according to HPV infection status. Receiver operating characteristic (ROC) curves were generated to assess diagnostic accuracy of each miRNA, and the area under the ROC curve (AUC) was calculated to measure discriminatory capacity. The best sensitivity/specificity pair was selected based on the maximum likelihood ratio. Univariate and multivariate logistic regression by odds ratio (OR) and 95% confidential interval (95% CI) were performed to assess predictors for cervical cancer diagnosis using the XLSTAT software (Addinsoft, New York, USA). All statistical tests were two-sided, and a *P* value ≤0.05 was considered statistically significant.

## Results

### HPV E6/E7 mRNA expression in cervical cancer tissues

Prior to investigating miRNA expression levels, we first examined HPV E6/E7 mRNA expression in 52 FFPE cervical cancer tissue samples and 50 FFPE normal control samples. Fifteen (28.8%) of the 52 FFPE cervical cancer tissue samples were negative for HR-HPV E6/E7 expression (termed HR-HPV E6/E7-negative), while 37 (71.2%) samples were positive (termed HR-HPV E6/E7-positive). We found that all 50 FFPE normal cervical control samples were negative for HPV E6/E7 mRNA expression (Table 1).

### MiRNA expression levels in cervical cancer and normal tissues

**Fig. 1:**
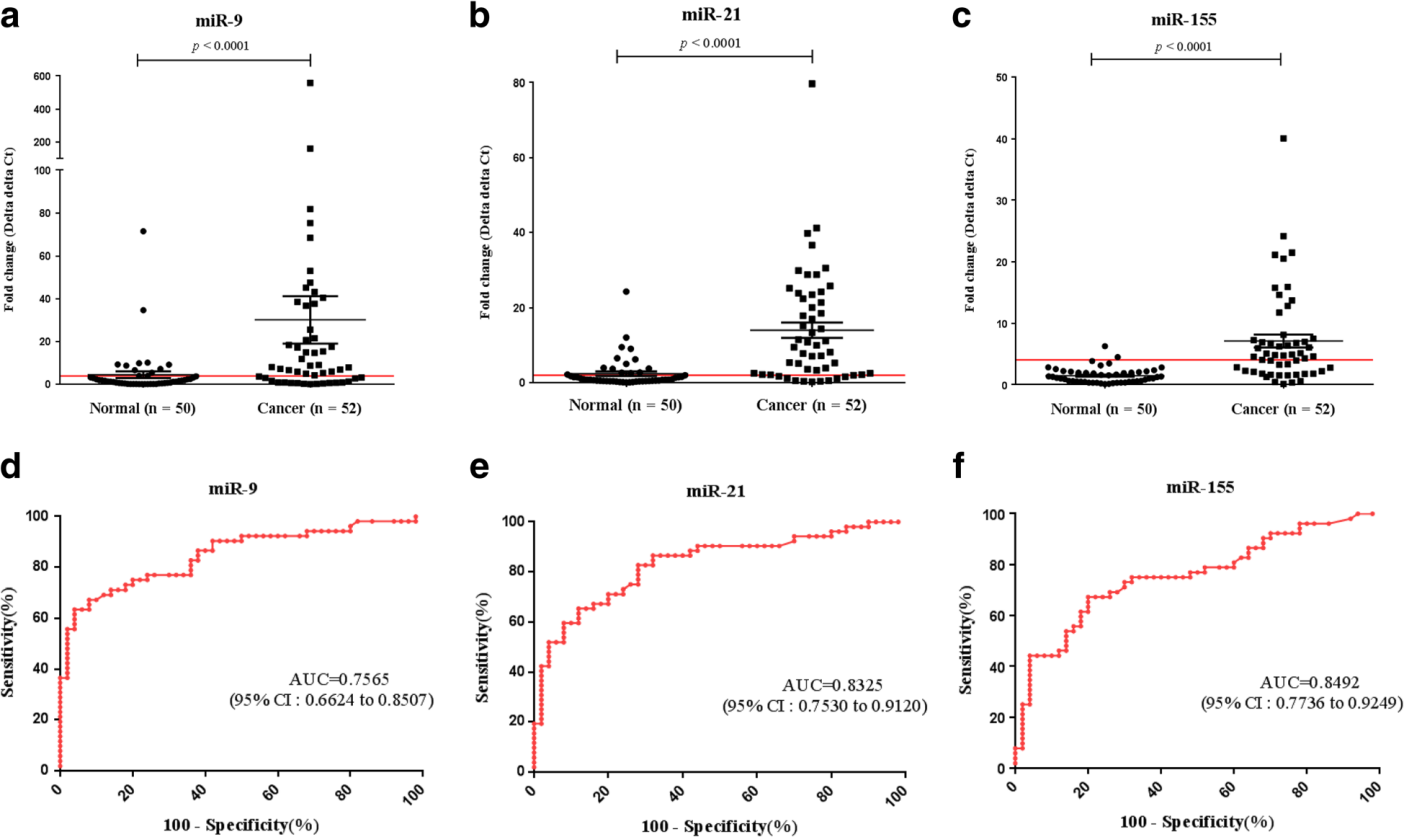
MiR-9, miR-21, and miR-155 expression levels in formalin-fixed paraffin-embedded (FFPE) cervical cancer and normal tissue samples. **a** MiR-9, **b** miR-21, and **c** miR-155 expression levels in 52 FFPE cervical cancer tissue samples were significantly different compared to that found in 50 FFPE normal cervical tissue samples (*P* < 0.0001 for all three comparisons). Receiver operating characteristic (ROC) curve analysis showed that **d** miR-9 had an area under the ROC curve (AUC) value of 0.7565 [95% confidence interval (CI) = 0.6624–0.8507], while **e** miR-21, and **f** miR-155 had AUC values of 0.8325 (95% CI = 0.7530–0.9120) and 0.8492 (95% CI = 0.7736–0.9249), respectively

Expression levels of miR-9, miR-21, and miR-155 were investigated in our 52 FFPE cervical cancer tissue samples and 50 FFPE normal cervical tissue controls. All three miRNAs were significantly up regulated in FFPE cervical cancer tissues compared to FFPE normal cervical tissues (*P* < 0.0001) (Fig. 1a-c). The AUC was 0.7565 [95% confidence interval (CI) = 0.6624–0.8507] in miR-9, 0.8325 (95% CI = 0.7530–0.9120) in miR-21, and 0.8492 (95% CI = 0.7736–0.9249) in miR-155, all of which indicate these miRNAs may be used as potential biomarkers for cervical cancer (Fig. 1d-f).

### Diagnostic value of miR-9, miR-21, and miR-155 miRNAs

To assess the potential diagnostic value of these three miRNAs, the performance characteristics sensitivity, specificity, positive predictive value, and negative predictive value were determined and evaluated. The cut-off values of miR-9, miR-21, and miR-155 as determined using the likelihood ratio, were 4.035, 1.975, and 3.880 respectively, for optimal sensitivity and specificity. The sensitivity of miR-9, miR-21, and miR-155 was 67.3% (95% CI = 52.9–79.7), 82.7% (95% CI = 69.7–91.8), and 65.4% (95% CI = 50.9–78.0) respectively, while the specificity was 80.0% (95% CI = 66.3–90.0) for miR-9, 72.0% (95% CI = 57.5–83.8) for miR-21, and 96.0% (95% CI = 86.3–99.5) for miR-155. Positive predictive values (PPVs) of miR-9, miR-21, and miR-155 were 77.8%, 75.4%, and 94.3% respectively, while the respective negative predictive values (NPVs) were 70.2%, 80.0%, and 71.6% (Table 2).

**Table 2:** Clinical cut-off values, sensitivity, and specificity of miRNAs

	Cut-off value	Sensitivity, % (95% CI)	Specificity, % (95% CI)	PPV, % (95% CI)	NPV, % (95% CI)	Likelihood ratio
miR-9	>4.035	67.3 (52.9–79.7)	80.0 (66.3–90.0)	77.8 (62.9–88.8)	70.2 (56.6–81.6)	3.4
miR-21	>1.975	82.7 (69.7–91.8)	72.0 (57.5–83.8)	75.4 (62.2–85.9)	80.0 (65.4–90.4)	3.0
miR-155	>3.880	65.4 (50.9–78.0)	96.0 (86.3–99.5)	94.3 (80.8–90.6)	71.6 (59.3–82.0)	15.9

*95% CI* 95% confidence interval, *PPV* positive predictive value, *NPV* negative predictive value

### MiR-9, miR-21, and miR-155 in HPV E6/E7-positive and -negative cervical cancer

**Fig. 2:**
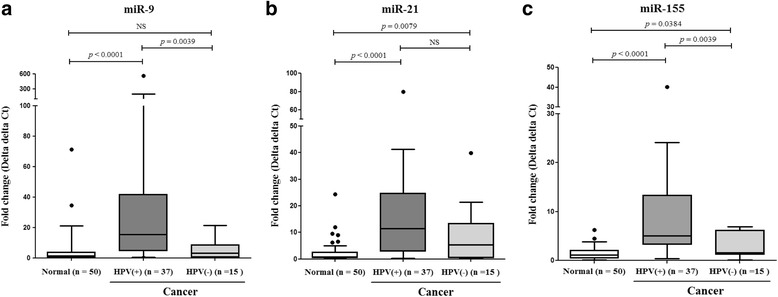
Box and whisker plots of comparisons between three miRNA expression levels in normal cervical tissues and cancer tissues with (+) or without (−) HPV E6/E7 mRNA expression. **a** MiR-9, **b** miR-21, and **c** miR-155 expression levels in high risk human papillomavirus (HR-HPV) E6/E7-positive cervical cancer tissues were significantly up regulated compared to that found in normal cervical tissue samples (*P* < 0.0001 for all three comparisons). MiR-21 and miR-155 expression levels in HR-HPV E6/E7-negative cervical cancer tissues were significantly up regulated compared to that found in normal cervical tissue samples (*P* = 0.0079 and *P* = 0.00384, respectively). NS, not significant

To investigate the expression of miR-9, miR-21, and miR-155 with cervical cancer cases that were HPV E6/E7 mRNA-positive or -negative for HPV E6/E7 mRNA, expression levels of the three miRNAs were analyzed in three groups: HPV E6/E7-positive cancer samples, HPV E6/E7-negative cancer samples, and normal samples. We found that all three miRNAs were significantly up regulated in HR-HPV E6/E7-positive cancer tissue samples compared to normal tissue samples (*P* < 0.0001), while miR-21 and miR-155 were up regulated in HPV E6/E7-negative cancer tissue samples compared to normal controls (*P* = 0.0079 and *P* = 0.0384, respectively). We found no significant difference in miR-9 expression levels between HR-HPV E6/E7-negative cancer samples and normal cervical samples (Fig. 2).

### Diagnostic value of miR-9, miR-21, and miR-155 miRNAs in conjunction with the HPV E6/E7 mRNA assay

The effectiveness of each three miRNA combined with HPV E6/E7 mRNA assay was investigated by analysis of AUC. In all cases of cervical cancer, the value of AUC was 0.8558 (95% CI 0.7773–0.9343), 0.8135 (95% CI 0.7257–0.9013), 0.8215 (95% CI 0.7349–0.9082), 0.8935 (95% CI 0.8243–0.9626) in HPV E6/E7, HPV E6/E7 + miR-9, HPV E6/E7 + miR-21, and HPV E6/E7 + miR-155, respectively. Especially, in HPV negative cervical cancer, miR-21 and miR-155 in conjunction with the HPV E6/E7 mRNA was shown increased AUC value of 0.7267 (95% CI 0.5776–0.8757) and 0.7000 (95% CI 0.5152–0.8646) respectively, compared to HPV E6/E7 assay (Table 3).

**Table 3:** Diagnostic values of miRNAs in conjunction with HPV E6/E7 for cervical cancer

	AUC^a^	95% CI	*P*-value
All cases			
HPV E6/E7	0.8558	0.7773–0.9343	<0.0001
HPV E6/E7 + miR-9	0.8135	0.7257–0.9013	<0.0001
HPV E6/E7 + miR-21	0.8215	0.7349–0.9082	<0.0001
HPV E6/E7 + miR-155	0.8935	0.8243–0.9626	<0.0001
HPV negative cases			
HPV E6/E7	0.5000	0.3321–0.6679	1.00
HPV E6/E7 + miR-9	0.6000	0.4284–0.7716	0.24
HPV E6/E7 + miR-21	0.7267	0.5776–0.8757	0.01
HPV E6/E7 + miR-155	0.7000	0.5152–0.8648	0.04

^a^ *AUC* an area under the ROC curve

### MiRNA predictors for diagnosing cervical cancer in HPV E6/E7-negative cases

We analyzed the following risk predictors of cervical cancer: age, HPV E6/E7 mRNA-expressing status, and miR-9, miR-21, and miR-155 expression. We found that the highest risk factor was HPV E6/E7 mRNA expression (OR = 244.4, 95% CI = 13.6–4376.4) followed by expression of miR-155 (OR = 41.7, 95% CI = 9.1–191.2), miR-21 (OR = 12.3, 95% CI = 4.8–31.7), and miR-9 (OR = 8.2, 95% CI = 3.3–20.3). Ages below 60 years were not a significant risk factor but being over the age of 60 years did confer a higher risk to cervical cancer (Table 4).

**Table 4:** The diagnostic utility of predictors for cervical cancer (univariate analysis)

	OR	95% CI	*P* value
Ages			
< 30 years	1		
31–40 years	1.4	0.4–5.1	0.580
41–50 years	1.7	0.4–6.5	0.442
51–60 years	3.5	0.8–16.4	0.109
> 60 years	35.2	3.6–344.2	0.002
HPV E6/E7			
negative	1		
positive	244.4	13.6–4376.4	<0.0001
miR-9			
negative	1		
positive	8.2	3.3–20.3	<0.0001
miR-21			
negative	1.0		
positive	12.3	4.8–31.7	<0.0001
miR-155			
negative	1.0		
positive	41.7	9.1–191.2	<0.0001

*OR* Odds ratio, *95% CI* 95% confidential interval

To investigate the predictive power for diagnosing cervical cancer in HPV E6/E7 mRNA negative cases, the odds ratio of the miRNAs was determined based on HPV E6/E7 mRNA expression using multivariate analysis. We found that miR-21 and miR-155 in HPV E6/E7-negative cervical cancer patients conferred a 7.0 (95% CI = 1.3–37.6) and 10.3 (95% CI = 1.5–70.7) fold risk to cervical cancer, respectively (Table 5).

**Table 5:** MiRNA predictors of cervical cancer according to HPV E6/E7 mRNA expression status in patients (multivariate analysis)

	OR	95% CI	*P* value
HPV positive			
miR-9	3.3	0.6–18.7	0.173
miR-21	1.8	0.3–11.1	0.515
miR-155	27.9	5.0–155.7	<0.0001
HPV negative			
miR-9	0.4	0.1–2.6	0.341
miR-21	7.0	1.3–37.6	0.024
miR-155	10.3	1.5–70.7	0.017

*OR* Odds ratio, *95% CI* 95% confidential interval

## Discussion

HPV DNA detection for cervical cancer screening is widely used as an early diagnostic guideline to prevent the progression of cervical cancer. Nevertheless, there is an ongoing need for studies investigating the molecular mechanisms related to cervical cancer carcinogenesis because most high-risk HPV infections that present without any symptoms go away within one to two years, and there have been reports of several HPV negative cases of cervical cancer [7–9, 25]. In our previous study, we found that 15 out of 100 FFPE cervical cancer tissue samples were HPV negative using an E6/E7 mRNA assay as well as testing for the HPV L1 genotype (data not shown).

Several oncogenic miRNAs are associated with cervical cancer tumorigenesis [17–23]. However, the results from those studies were not comprehensively evaluated using clinical specimens, and those studies have not tested for an association between miRNA expression levels and HR-HPV E6/E7 mRNA expression in clinical specimens. The aim of this study was to explore the potential clinical relevance of miR-9, miR-21, and miR-155 by investigating their expression levels in 52 FFPE cervical cancer tissue samples and in 50 FFPE normal cervical tissue samples. Evidence of association between these three miRNAs and HR-HPV E6/E7 mRNA expression was also investigated.

All three miRNAs (miR-9, miR-21, and miR-155) showed significantly higher expression in cervical cancer tissues compared to that found in normal cervical tissues (*P* < 0.0001) (Fig. 1). This finding supports the possibility that these three miRNAs may be implicated in cervical cancer development in clinical samples. Although four previous studies using cervical cancer cell lines and clinical samples found that miR-21, miR-155, and miR-9 were up regulated in cervical cancer, they did not validate these three miRNAs in terms of diagnostic value [13, 18, 26, 27]. This study is the first to assess these miRNAs as putative biomarkers and their possible discriminatory capacity in FFPE tissues.

Differences in expression levels of the three miRNAs between HR-HPV E6/E7-positive cervical cancer tissue samples and HR-HPV E6/E7–negative cervical cancer tissue samples showed the strongest association was between miR-9 expression and HR-HPV E6/E7-positive cancer cases compared to that found with the other miRNAs (Fig. 2). Similarly, Weijun Liu et al. found miR-9 and HPV E6 caused increased cell motility by down regulating follistatin like 1 (FSTL1) and activated leukocyte cell adhesion molecule (ALCAM) mRNAs, both of which are involved in cell migration [28].

Both miR-21 and miR-155 had reported other mechanism related to immune response as well as HR-HPV E6/E7 expression. Bumrungthai et al. found that miR-21 is correlated with increased expression of α-smooth muscle actin (α-SMA) and interleukin 6 (IL-6) and decreased expression of PDCD4 in cell proliferation and initiates inflammation-associated carcinogenesis via nuclear factor kappa-light chain-enhancer of activated B cells (NF-kB) and interleukin-6 (IL-6) signaling pathways in colon and cervical cancer cells and Asangani et al. found miR-21 down-regulates PDCD4 in colon cancer and functions as stimulating invasion, intravasation, and metastasis [21, 22, 29]. For miR-155, Guoying Lao et al. found that miR-155 regulates LKB1 expression, which functions as embryonic polarity, metabolism, and cell growth and up-regulation of miR155 promotes proliferation of cervical cancer cells [23].

In terms of diagnostic value, miR-21, and miR-155 expression in combination with the HPV E6/E7 mRNA assay may be useful in the diagnosis of cervical cancer independent of HPV infection because these miR-21 and miR-155 may have the discriminatory power to detect HPV negative cervical cancer cases (Table 3). In particular, miR-21 was independent of HPV status being consistently up regulated in cervical cancer (Fig. 2). We analyzed the odds ratios to assess the effects of the predictors age, HPV E6/E7 mRNA expression, and expression of miR-9, miR-21, and miR-155 on cervical cancer compared to normal controls. While we found that all of the predictors were significantly associated with cervical cancer, miR-21 and miR-155 expression were identified as predictors for high risk in HPV negative cervical cancer tissues compared to normal cervical tissues (Tables 4 and 5).

Some studies have investigated miRNAs for association with HPV infection as potential diagnostic and prognostic indicators. Xiaohong Wang et al. revealed that miR-92a and miR-378 expression was associated with cancer progression in HPV positive tissue samples [30]. Similarly, we found that miR-155 overexpression is associated with increased risk of cervical cancer in HPV E6/E7 mRNA positive tissues. Moreover, miR-21 and miR-155 overexpression in HPV E6/E7 mRNA negative tissue samples could complement approaches for cervical cancer diagnosis and prediction of progression.

## Conclusions

Our findings showed that miRNA RT-qPCR assays for specific miRs may be useful tools in the diagnosis of cervical cancer and especially, HPV negative cases of cervical cancer. In addition, these findings are important towards determining the possible role of miRNA expression in cervical cancer development, and the relationship between miRNA and HPV infection. Further study is needed in pre-cancer lesions to understand the role of miRNAs in tumor carcinogenesis, and more tests using normal and cervical cancer samples will be necessary to clearly demonstrate the potential utility of these assays for cervical cancer screening and diagnosis.

## Abbreviations

95% CI: 95% confidential interval
ALCAM: Activated leukocyte cell adhesion molecule
AUC: Area under the ROC curve
FFPE: Formalin-fixed paraffin-embedded
FSTL1: Follistatin like 1
GAPDH: Glyceraldehyde-3-phosphate dehydrogenase
HR-HPV: High risk human papillomavirus
IL-6: Interleukin-6; LKB1: Liver kinase B1
miRNA: microRNA
NPV: Negative predictive value
OR: Odds ratio
PPV: Positive predictive value
pRb: Retinoblastoma protein
ROC: Receiver operating characteristic
α-SMA: α-smooth muscle actin
